# Core shell lipid-polymer hybrid nanoparticles with combined docetaxel and molecular targeted therapy for the treatment of metastatic prostate cancer

**DOI:** 10.1038/s41598-017-06142-x

**Published:** 2017-07-19

**Authors:** Qi Wang, Heba Alshaker, Torsten Böhler, Shyam Srivats, Yimin Chao, Colin Cooper, Dmitri Pchejetski

**Affiliations:** 1 School of Medicine, University of East Anglia, Norwich, UK; 2 Department of Pharmacology and Biomedical Sciences, Faculty of Pharmacy and Medical Sciences, University of Petra, Amman, Jordan; 3 Department of Surgery and Cancer, Imperial College London, London, UK; 4 School of Medicine, University of California San-Francisco, San-Francisco, CA USA; 5 School of Chemistry, University of East Anglia, Norwich, UK

## Abstract

Many prostate cancers relapse after initial chemotherapy treatment. Combining molecular and chemotherapy together with encapsulation of drugs in nanocarriers provides effective drug delivery and toxicity reduction. We developed core shell lipid-polymer hybrid nanoparticles (CSLPHNPs) with poly (lactic-co-glycolic acid) (PLGA) core and lipid layer containing docetaxel and clinically used inhibitor of sphingosine kinase 1 (SK1) FTY720 (fingolimod). We show for the first time that FTY720 (both free and in CSLPHNPs) re-sensitizes castrate resistant prostate cancer cells and tumors to docetaxel, allowing a four-fold reduction in effective dose. Our CSLPHNPs showed high serum stability and a long shelf life. CSLPHNPs demonstrated a steady uptake by tumor cells, sustained intracellular drug release and *in vitro* efficacy superior to free therapies. In a mouse model of human prostate cancer, CSLPHNPs showed excellent tumor targeting and significantly lower side effects compared to free drugs, importantly, reversing lymphopenia induced by FTY720. Overall, we demonstrate that nanoparticle encapsulation can improve targeting, provide low off-target toxicity and most importantly reduce FTY720-induced lymphopenia, suggesting its potential use in clinical cancer treatment.

## Introduction

In the Western world, prostate cancer is now the most commonly diagnosed noncutaneous cancer in men and the second leading cause of cancer mortality^1^. Hormone therapy is offered for patients with locally advanced or metastatic disease, but many tumors relapse. Docetaxel is a first line chemotherapy offered to these patients, however, it only extends survival for a median period of less than three months^2^. Recently published GETUG-AFU 15^3^ and STAMPEDE^4^ clinical trials revived the interest in docetaxel showing that its early administration together with androgen deprivation therapy could largely improve the overall survival. New ways of re-sensitizing prostate cancer to docetaxel would present a significant therapeutic benefit.

Sphingosine kinase 1 (SK1) is a proto-oncogenic enzyme that is highly expressed in human prostate tumors^5^ and correlates with prostate cancer cell chemoresistance^6^. Silencing SK1 sensitizes prostate tumors to docetaxel chemotherapy^7, 8^ and can potentially provide a new chemotherapeutic modality for patients with prostate cancer resistant to docetaxel. FTY720 is a functional antagonist of sphingosine-1-phosphate (S1P) receptors and an inhibitor of SK1^9^. It is clinically used to treat multiple sclerosis^10^ by inducing lymphopenia and T-cell sequestration to lymph nodes^9^, which is the main obstacle for FTY720 use in cancer patients. The main adverse effects of docetaxel chemotherapy are non-hematologic toxicities and febrile neutropenia^11^.

Encapsulation of drugs in nanocarriers that target cancer cells is an effective method of co-delivery of drug combinations and toxicity reduction. Liposomes and polymers are highly biocompatible and are commonly used for the synthesis of drug-containing nanoparticles^12^. Liposome surface can be functionalized readily with hydrophilic polymers, such as poly(ethylene glycol) (PEG), prolonging plasma half-life and providing links to tumor targeting molecules^13^. Polymer nanoparticles show strong structural stability and are able to encapsulate drugs with high capacity^14^. Hybrid lipid-polymer nanoformulations combine the advantages of both models, allowing controlled drug release and enhanced bio-functionality^15^. Recent evidence shows that nanoformulations provide significant advantage to prostate cancer molecular therapies and chemotherapies allowing improvement in drug delivery and imaging^16–18^. This is particularly true for docetaxel where nanoformulations outperform systemic therapy^19, 20^. Abraxane, an albumin simple nanoparticle bound paclitaxel is currently licensed for use in breast, lung and pancreatic cancers.

In this study, we have tested whether encapsulation of SK1 inhibitor FTY720 (fingolimod) and docetaxel into nanoparticles may reduce systemic absorption and provide targeted delivery to the tumors. Core shell lipid-polymer hybrid nanoparticles (CSLPHNPs) with poly (lactic-co-glycolic acid) (PLGA) core and lipid layer containing docetaxel and FTY720 showed high serum stability, a long shelf life, a steady uptake by tumor cells and sustained intracellular drug release. Our data show that FTY720 (both free and in CSLPHNPs) re-sensitized castrate resistant prostate cancer cells and tumors to low doses of docetaxel. In mice, CSLPHNPs showed excellent tumor targeting and significantly lower side effects compared to free drugs, notably reversing lymphopenia induced by FTY720.

## Methods

### Synthesis and characterization of CSLPHNPs

PLGA nanoparticle cores were prepared by emulsion-solvent evaporation technique^21^. Briefly, PLGA and docetaxel 10:1 wt% were dissolved completely in acetone. The entire solution was emulsified into 2% aqueous solution of 80% poly vinyl alcohol (PVA) by slow injection with homogenization. This mini-emulsion was then added to a 0.2% PVA solution with rapid mixing overnight to evaporate any residual acetone. Nanoparticle-size fraction was recovered by ultracentrifugation at 10,000, 20,000 and 80,000 × *g*. A mixture of phospholipids (phosphatidylcholine and 1,2-distearoyl-sn-glycero-3-phosphoethanolamine-N-[methoxy(polyethylene glycol)-2000] (18:0 PEG2000 PE)), cholesterol and FTY720 were dissolved in chloroform, and then a lipid film was formed in a round bottom flask under reduced pressure using a vacuum rotary evaporator. An aqueous solution of the smallest size fraction of PLGA nanoparticles was added to the film. The resulting suspension was extruded through a 200 nm membrane using a hand held extruder (Avanti, Alabaster) to create the lipid vesicles. The resulting hybrid lipid-polymer nanoparticles were collected by Amicon centrifugal filters (MWCO 100 K Da) and washed twice with distilled water.

### Morphology and size measurements of CSLPHNPs

The surface morphology of the nanoparticles was studied using scanning electron microscopy (JEOL Ltd, UK). The CSLPHNPs were dried on an aluminum stub, coated with gold to obtain a uniform layer of particle and dried overnight. For dynamic light scattering (DLS) measurement, the size distribution and zeta potential of the nanoparticles were estimated using a Zetasizer Nano ZS90 (Malvern Instruments, Malvern, UK) with 90° optics and a He-Ne Laser. Nanoparticle physical structure was examined using freeze-fracture scanning electron microscopy (FF-SEM). The sample solution was frozen at −210 °C, fractured at −100 °C, sputter coated with platinum and then imaged using an FEI Nova NanoSEM 450 FEG scanning electron microscope (FEI, Eindhoven, The Netherlands).

### Liquid chromatography-tandem mass spectrometry analysis

To study the release profile of CSLPHNPs *in vitro*, nanoparticles were suspended in 1 ml of pH5/pH 7.4 buffer and were placed in an Amicon dialysis device (MWCO 1000 Da) suspended in 14 ml pH5/pH 7.4 buffer. After stipulated time points 2 ml aliquots of water was extracted and replaced with fresh water (pH 5). The dialysis device was incubated at 37 °C with gentle shaking. Aliquots were extracted from the incubation medium at predetermined intervals, and released drug was quantified by an Agilent6400 Triple Quadrupole LC-MS/MS system. Samples were applied to a Ascentis Express C18 reverse phase column (50 × 2.1 mm, 2.7 µm) and eluted using an 80:20 acetonitrile:isopropanol (ACN:IPA), water, formic acid gradient (flow rate 0.4 ml/min).

### Cell lines and cell culture

Androgen insensitive prostate cancer cell lines (PC-3 and DU145) were obtained from DSMZ (Braunschweig, Germany). Cells were maintained in tissue culture flasks or plastic dishes in a humidified atmosphere of 5% CO_2_ at 37 °C using RPMI 1640 supplemented with 10% heat-inactivated fetal bovine serum (FBS)  (Sigma‐Aldrich, UK), 50 U/ml penicillin, 50 μg/ml streptomycin and 2 mM glutamine (Sigma‐Aldrich, UK). Cell lines were routinely verified by morphology and growth curve analysis. All experiments were conducted in the absence of serum. Cells were seeded to be 80% confluent by the end of treatment and were treated as indicated in figures’ legends. Cell lines were kept in culture for up to 30 passages.

### Cell viability

Cells were grown in 96-well plates, starved, and exposed to different treatments as indicated in figure legends. Cellular viability was measured using the 3-(4,5-dimethylthiazol-2-yl)-2,5-diphenyl tetrazolium bromide (MTT; 5 mg/ml) colorimetric assay as already described^22^.

### Fluorescent microscopy and quantification of nanoparticle uptake

Prostate cancer cells were seeded onto 6 well plates and treated with CSLPHNPs loaded with Rhodamine B for up to 72 h. After incubation, the cells were washed three times with phosphate buffered saline (PBS) and fluorescent imaging was performed using fluorescent microscope (Carl Zeiss, USA). The fluorescent intensities in cells were quantitatively analyzed by ImageJ.

### Sphingosine kinase 1 activity assay

SK1 assay was performed using radiolabeling as previously described^22–24^, in conditions favoring SK1 activity and inhibiting SK2 activity.

### Animal study

Animal study was performed as previously described^6, 25, 26^. Briefly, subcutaneous human prostate cancer xenografts were established in NOD scid gamma (NSG) immunodeficient male mice by subcutaneous injection of 10^6^ PC-3 cells. Three weeks after implantation, mice were randomized into treatment groups (n = 7/group) and treated twice a week for two weeks with: i.v. tail vein injections of: saline, 5 mg/kg FTY720 + 5 mg/kg docetaxel, empty CSLPHNPs, CSLPHNP (5 mg/kg FTY720 + 5 mg/kg docetaxel and labelled with CF488). One day after the last treatment, all mice were euthanized and blood collected for white cell count (WCC) measurement. Mice, tumors and individual organs were weighed and primary tumors were then processed for analysis of SK1 activity (radiolabeling) as described above. Primary tumors, livers, spleens and kidneys were fixed with 4% paraformaldehyde and fluorescent images were obtained using a stereo microscope and quantified using ImageJ. Animal studies were performed under the Home Office license and carried out in in accordance with the institutional guidelines and regulations for animal welfare (University of East Anglia) and NC3Rs (Replacement, Reduction and Refinement) guidelines. The experiments were carried out in accordance with the protocols section 19B of the Home Office license and were approved by the University of East Anglia animal welfare committee.

### Statistical analysis

Data are presented as the mean values of at least three independent experiments normalized to control ± standard error of the mean (SE) calculated using OriginPro8.0. Statistical significance between two groups was conducted by unpaired Student’s t test. Statistical significance between three or more groups was conducted by analysis of variance (ANOVA). *p*-value of < 0.05 is considered statistically significant.

## Results

### Nanoparticle synthesis and characterization

**Figure 1:**
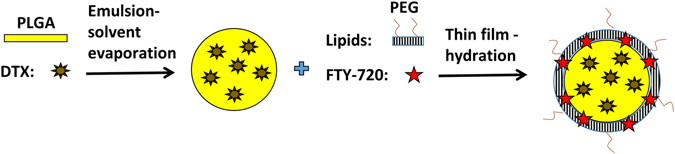
Scheme of CSLPHNPs synthesis and drug loading. Schematic representation of the preparation of CSLPHNPs with a multiple layer-by-layer structure. Briefly, organic solution containing PLGA and docetaxel was emulsified into an aqueous solution of PVA by slow injection with homogenization. After removing of any residual solvent, the nanoparticle-size fraction was recovered by ultracentrifugation. The smallest size fraction of PLGA nanoparticles aqueous solution was added to the lipid film containing phospholipids, cholesterol and FTY720 mixture to form CSLPHNPs. The resulting CSLPHNPs were extruded through a 200 nm membrane, purified and collected by Amicon centrifugal device. DTX, docetaxel.

**Figure 2:**
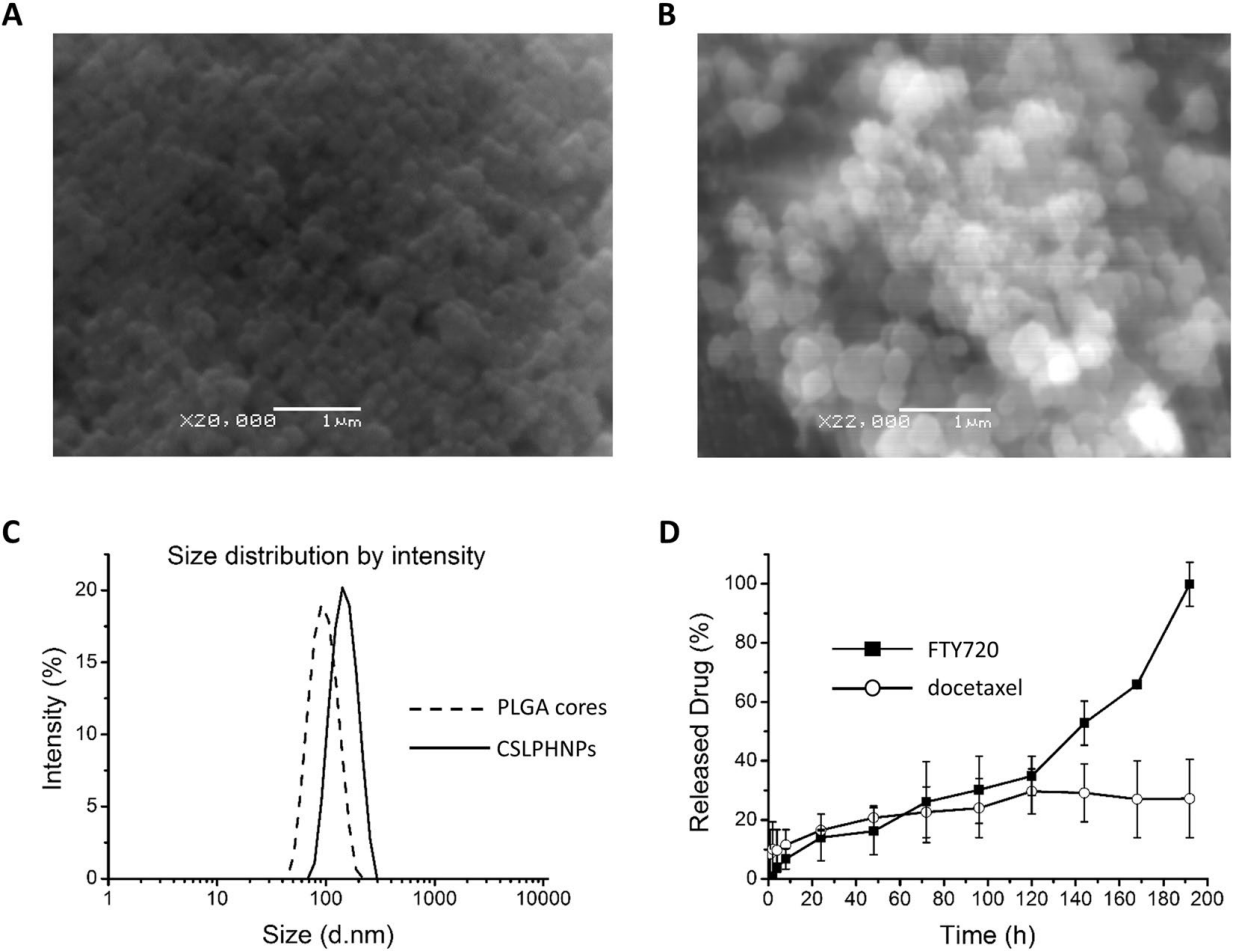
Characterization of CSLPHNPs. Scanning electron microscopy image of (**A**) PLGA cores and (**B**) CSLPHNPs; 20,000/22,000X magnification (Scale bar: 1 µm). (**C**) Dynamic light scattering (DLS) measurement of the size distribution of PLGA cores-dash line, CSLPHNPs-solid line. (**D**) Temporal release of FTY720 (black squares) and docetaxel (white circles) at pH 5 was measured by liquid chromatography-tandem mass spectrometry (LC-MS/MS). Points, mean of three experiments performed in triplicate. Bars, SE.

To synthesize CSLPHNPs we have combined emulsion evaporation of PLGA forming polymer core and the thin film hydration forming a lipid shell (Fig. 1). The solid polymeric core acts as a scaffold to encapsulate docetaxel. The lipid shell contains FTY720 and envelopes the core preventing internal drug leakage at plasma pH (Figure S1), while releasing the drugs at lysosomal pH (Fig. 2D).

CSLPHNPs were designed to fit the 40–200 nm size range for enhanced delivery to tumor leaky vessels according to the theory of enhanced permeability and retention^27^. Scanning electron microscopy shows that CSLPHNPs were dispersed, with a well-defined spherical shape (Fig. 2A,B). Freeze-fracture scanning electron microscopy (FF-SEM) demonstrated that CSLPHNPs exhibited a core shell structure (Figure S2).

Particle sizes, polydispersity index (PDI) and zeta potentials of PLGA cores and CSLPHNPs are shown in Figures S3 and S4. The sizes of PLGA cores and CSLPHNPs were 88.4 ± 1.7 nm and 141.5 ± 1.2 nm, respectively (Figs 2C and S4). Due to the PLGA terminal carboxyl acid, PLGA nanoparticles showed an average negative surface charge of −29.9 mV in PBS (Figure S4).

### Drug encapsulation and release

Nanoparticle translation into clinic is limited by their stability in body environments. CSLPHNPs had high colloidal stability in various biological media showing no significant change in size and PDI (Figure S3), suggesting that they could be stored with little or no aggregation. In pure FBS and 10% human plasma solution CSLPHNPs showed an initial ~15 nm increase in size and 0.20 increase in PDI, after which they maintained size and stability throughout the five-days study (Figure S3), suggesting that plasma protein binding was not a significant modifying factor, possibly due to the stable core-shell structure and the steric repulsion of the PEG chain because of its hydrodynamic diameter.

High drug loading and controlled release of contents are two important advantages of CSLPHNPs. The encapsulation of docetaxel and FTY720 in CSLPHNPs was 10% and 70%, respectively, as confirmed by liquid chromatography-tandem mass spectrometry (LC-MS/MS). The low encapsulation efficiency of docetaxel was probably due to the size selection process of PLGA core synthesis. *In vitro* release profiles of FTY720 and docetaxel were pH dependent with a “lysosomal” pH 5 (Fig. 2D) showing a quicker release than “serum” pH 7.4 (Figure S1). At pH 5, the release pattern of both drugs was initially similar, followed by a surge in FTY720 at 96 h indicating disintegration of the lipid outer layer, reaching 100% at 192 h.

### Testing of nanoparticles in cell and animal models of prostate cancer

**Figure 3:**
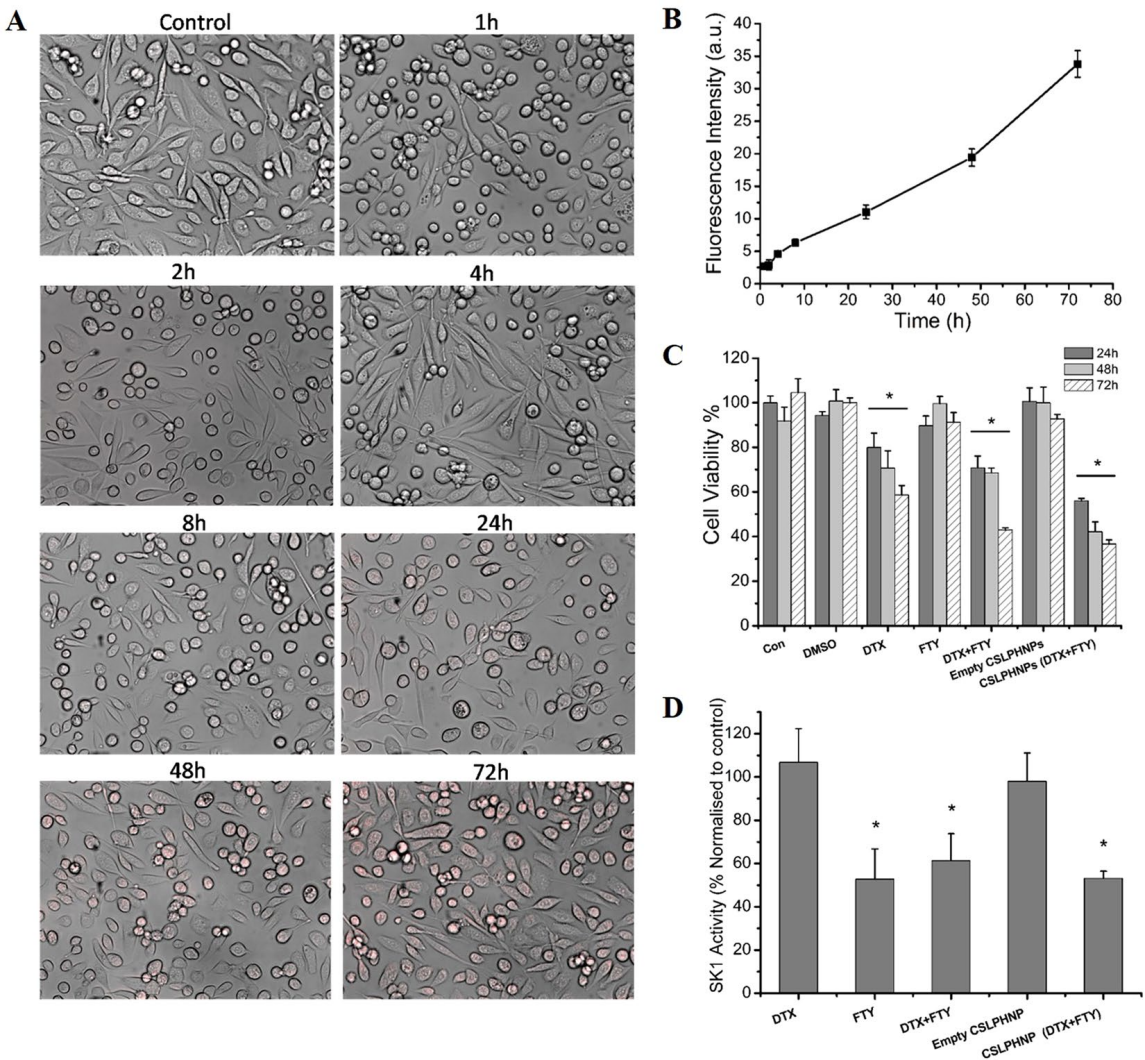
*In vitro* assessment of CSLPHNPs. Hormone-refractory metastatic prostate cancer PC-3 cells were treated with free drugs or CSLPHNPs for 72 hours. (**A**) Fluorescent/phase contrast images of PC-3 cells after incubation with CSLPHNPs containing Rhodamine B. (**B**) CSLPHNPs uptake assessed by fluorescent microscopy (**A**) was quantified by ImageJ. (**C**) Cytotoxicity of CSLPHNPs was assessed using the MTT assay. Columns, mean of three experiments performed in triplicate. Data is expressed as mean (n = 3) ±SE. *p < 0.05 versus control. **D)** PC-3 cells were treated for 48 h as indicated in figure and SK1 activity was measured by radiolabeling. Data is expressed as percentage relative to the control. Columns represent the mean of n = 3 ± SE. *p < 0.05 versus control.

CSLPHNPs were readily taken by prostate cancer cells. Fluorescent microscopy showed a rapid time-dependent accumulation of rhodamine B-loaded CSLPHNPs within prostate cancer cells (Figs 3A,B and S5). In PC-3 and DU145 cells the most effective molar ratio of FTY720:docetaxel is 5 µM:5 nM, respectively, and both drugs show additive effect (Figs 3C and S6). While empty CSLPHNPs exhibited no significant cytotoxicity in PC-3 cells, CSLPHNPs containing same amount of FTY720/docetaxel were 15%, 27% and 6% more cytotoxic than free drugs at the 24, 48, and 72 h, respectively. In DU145 prostate cancer cells, CSLPHNPs induced loss of cell viability similar to free drugs (Figure S6). FTY720 is a known SK1 inhibitor^9^ and Fig. 3D shows that all FTY720 containing therapies including CSLPHNPs have significantly reduced SK1 activity in a similar fashion, while docetaxel alone did not lead to significant changes.

**Figure 4:**
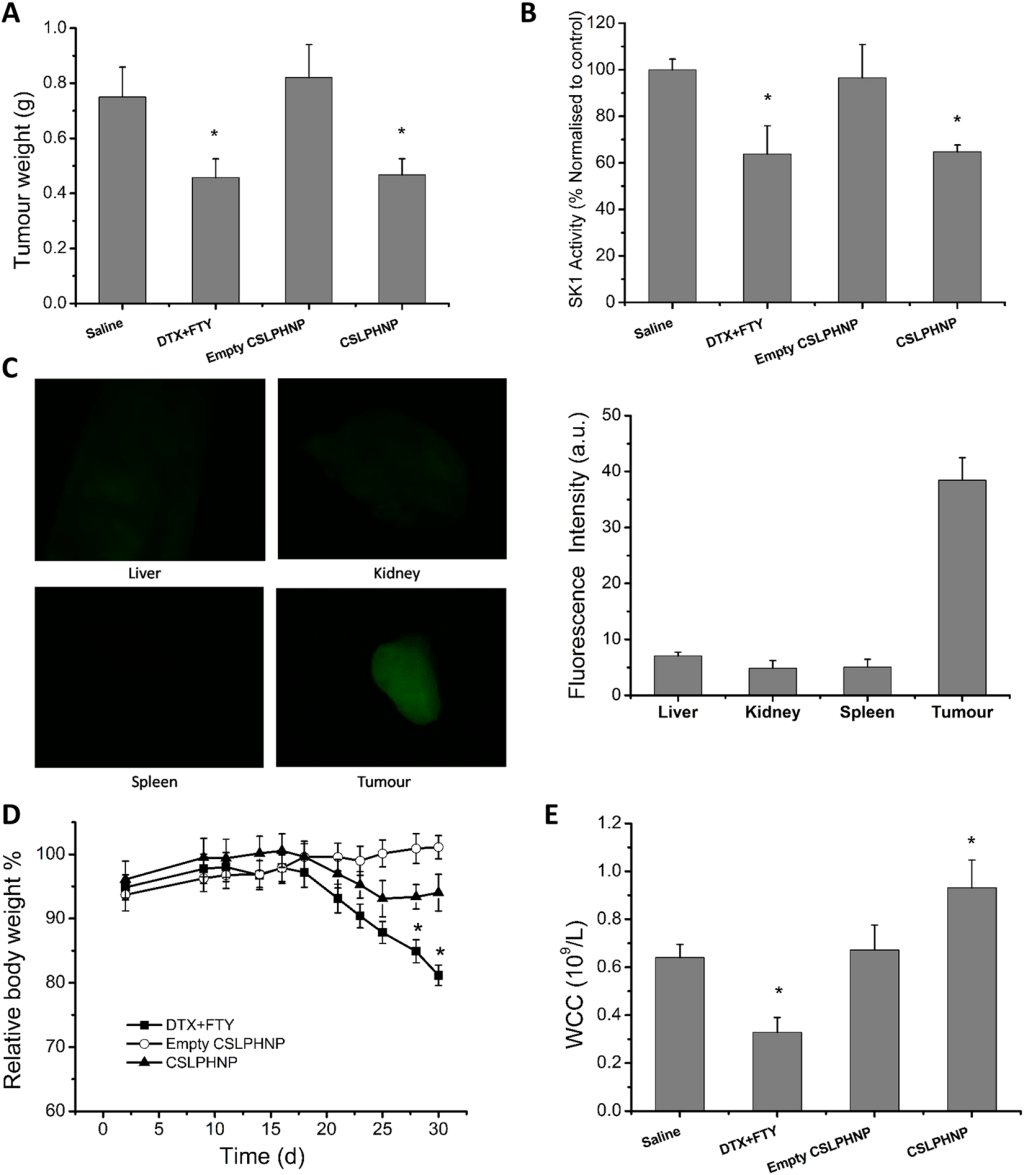
*In vivo* assessment of CSLPHNPs. NSG immune deficient mice were injected with 1 × 10^6^ PC-3 cells and tumors were grown for 3 weeks. Mice were randomized in groups (n = 7) according to treatment as shown in figure, and treated twice a week for two weeks. CF488 fluorophore labelled CSLPHNPs were injected in the last treatment cycle. (**A**) Tumor weight at week five. (**B**) Intratumoral SK1 activity. (**C**) Fluorescent microscopy of mouse organs and primary tumors. Graph (on the right) indicates the levels of fluorescence in each organ quantified using ImageJ. (**D**) Relative body weight. (**E**) White cell count (WCC). Data is presented as mean ± SE, (n = 7), *p < 0.05 versus control.

To investigate the *in vivo* effects of CSLPHNPs we used an animal model of human prostate tumors. NSG immunodeficient mice were inoculated with PC-3 cells. Tumors were grown for three weeks and then treated for two weeks with saline, free drug combination (5 mg/kg FTY720 + 5 mg/kg docetaxel), empty CSLPHNPs labelled with CF488 fluorophore or CSLPHNPs (5 mg/kg FTY720 + 5 mg/kg docetaxel + CF488). In the saline and the empty CSLPHNPs groups, tumors rapidly grew reaching 0.75 ± 0.11 g and 0.82 ± 0.12 g, respectively, by day 30, while free therapies and CSLPHNP have significantly reduced the tumor weight to 0.46 ± 0.07 g and 0.47 ± 0.06 g, respectively (Fig. 4A). Similar to *in vitro* studies, all treatments containing FTY720 have significantly reduced tumor SK1 activity (Fig. 4B). CSLPHNPs showed excellent tumor targeting capability with ~40% of CSLPHNPs localized in the tumor (Fig. 4C), suggesting a high selection towards unorganized tumor vessels^27^ with a minimal penetration into other tissues.

Chemotherapy-induced whole body toxicity is of utmost clinical importance as it is the key limiting factor to the administration of effective chemotherapy doses in cancer patients. In NSG mice, free FTY720 + docetaxel therapy, while having good anti-tumor efficacy (Fig. 4A) induced a 20% reduction in total body weight (Fig. 4D), significantly reducing liver size (Figure S7) and general mouse wellbeing. In contrast, CSLPHNPs did not affect body weight (Fig. 4D) or liver weight (Figure S7) demonstrating the best combination between efficacy (similar to free drugs) and lack of toxicity (similar to chemotherapy-free nanoparticles).

The major obstacle for FTY720 use in cancer patients is significant lymphopenia induced by this drug due to T-cell sequestration to lymph nodes^9^. Hematological assessment showed that free FTY720 has significantly reduced WCC after two weeks of administration (Fig. 4E). In contrast, CSLPHNPs containing FTY720 showed no decrease in WCC, effectively overcoming FTY720-induced lymphopenia.

## Discussion

In this paper, we addressed an important clinical problem of overcoming docetaxel chemoresistance in prostate cancer patients. This could be achieved through the use of molecular targeted therapy as a sensitizer and nanoparticle encapsulation to reduce toxicity and improve tumor targeting (Fig. 1).

Docetaxel on its own was not effective in the PC-3 or DU145 cells (Figs 3C and S6) therefore representing a model of chemoresistance. We have then combined it with an SK1 inhibitor FTY720 and demonstrated an effective chemosensitization (Figs 3C and S6). This is a new combination of a molecular sensitizer and chemotherapy based on our previous studies implicating SK1 in prostate cancer chemoresistance^6, 26, 28^. Our previous studies show that 20 nM docetaxel is required for successful targeting of prostate cancer cells^6^, therefore a dose of 5 nM used in this study represents a 4-fold reduction in the effective dose^25^.

The synthesized CSLPHNPs possessed the required properties for the tumor targeting according to the enhanced permeability and retention^27^, such as 40–200 nm size and narrow PDI (Figs 2, S1 and S3). The CSLPHNPs had the benefit of prolonged and lysosomal pH-specific drug release (Figs 2 and S1). In contrast to FTY720, only around 30% of docetaxel was released into the medium, which indicates, that most of the docetaxel was still under protection of the polymeric core. It is, however, possible that in cells PLGA core may be broken down through enzymatic degradation^29, 30^ leading to docetaxel release. Docetaxel has a short circulation half-life of ~17 hours^31^, FTY720 has a long half-life of 6–9 days^32^ and PEG functionalized liposome nanoparticles have half life time >5 days^33^. It is likely that FTY720/docetaxel encapsulation in CSLPHNPs allows stabilization of their intracellular release (Fig. 2D), providing an increased cytotoxic benefit.

CSLPHNPs are readily taken up by prostate cancer cells and have excellent anticancer properties exceeding those of free drugs (Figs 3, S5 and S6). FTY720 is a known SK1 inhibitor^9^ and Fig. 3D shows that all FTY720 containing therapies including CSLPHNPs have significantly reduced SK1 activity in a similar fashion, while docetaxel has induced an insignificant increase. Taken together our data suggest that FTY720 potentiates docetaxel effect through downregulation of SK1, and that the combined nanoformulation improves chemotherapy efficacy possibly through localized release of higher concentration of both drugs inside the cells.

*In vivo*, 5 mg/kg FTY720 sensitized human prostate tumors grown in NSG mice to 5 mg/kg docetaxel and CSLPHNPs had a similar effect (Fig. 4A). Previously we have shown that at least 20 mg/kg docetaxel is required for successful *in vivo* prostate tumor treatment^6^, therefore our current findings represent a considerable clinical advantage. CSLPHNPs had a high tumor targeting capability with a minimal penetration into other tissues (Fig. 4C). This is achieved through two main mechanisms. First, according to enhanced permeability/retention theory, nanoparticles of ~100 nM can extravasate only through “leaky” tumor vessels, but not through normal vasculature. Therefore, they cannot infiltrate normal tissues including liver and kidneys, which have fenestrations only <40 nm. Second, covering with glucosamine directs the nanoparticles to the tissues with a rapid metabolic turnover, including tumors.

This specific targeting has led to a significant reduction in toxicity as evidenced by increase in total body weight (Fig. 4D) and liver size (Figure S7) in comparison to free drugs. The major obstacle for FTY720 use in cancer patients is significant lymphopenia^9^. Hematological assessment showed that free FTY720 has significantly reduced WCC, while CSLPHNPs showed no decrease (Fig. 4E). When free FTY720 is administered systemically, it antagonizes the lymphocyte S1P receptors in the lymph nodes^34^. S1P levels are higher in the peripheral blood than in lymph nodes and serve as the driving force of lymphocyte egress to the periphery. With S1P receptors blocked, lymphocytes remain in the lymph nodes causing peripheral lymphopenia^35–37^. Here we show that encapsulation of FTY720 and its targeted delivery to tumor cells bypassing other tissues allows effective preservation of WCC in treated animals. This is the first study demonstrating a mechanism to overcome FTY720-induced lymphopenia, allowing its potential use in cancer patients. Our data demonstrate a clear advantage of CSLPHNPs over free FTY720+ docetaxel systemic therapy, specifically due to tumor targeting and reducing unwanted side effects.

In summary, in the current study, we have identified a new combination therapy for castrate resistant prostate cancer containing FTY720 and docetaxel. We have developed a CSLPHNPs formulation for delivering this new combination therapy in prostate cancer cells and tumors. We have shown that FTY720 re-sensitizes prostate cancer cells and tumors to low dose docetaxel and nanoparticle encapsulation allows a steady sustained release of both drugs combined with an excellent targeting capability, biocompatibility and very low toxicity in comparison to free therapies. An important novelty of this study is the development of a successful approach to overcome FTY720-induced lymphopenia, allowing its potential use in cancer patients. Collectively, these results provide the first evidence of improved safety and efficacy of nanoparticle encapsulated tumor targeted FTY720 and docetaxel combination chemotherapy for treatment of hormone-resistant prostate cancer.

## Electronic supplementary material

supplementary figures
