# Supplementary material for: Core shell lipid-polymer hybrid nanoparticles with combined docetaxel and molecular targeted therapy for the treatment of metastatic prostate cancer
Source: Sci Rep. 2017 Jul 19;7:5901. doi: 10.1038/s41598-017-06142-x (PMC5517417; doi:10.1038/s41598-017-06142-x)
Supplement: Supplementary file 1 — supplementary figures [file 41598_2017_6142_MOESM1_ESM.doc]

**Supplementary Figures**

**Core shell lipid-polymer hybrid nanoparticles with combined docetaxel and molecular targeted therapy for the treatment of metastatic prostate cancer**

Qi Wang^1*^, Heba Alshaker^1,2^, Torsten Böhler^3^, Shyam Srivats^4^, Yimin Chao^5^, Colin Cooper^1^, Dmitri Pchejetski^1*^

**
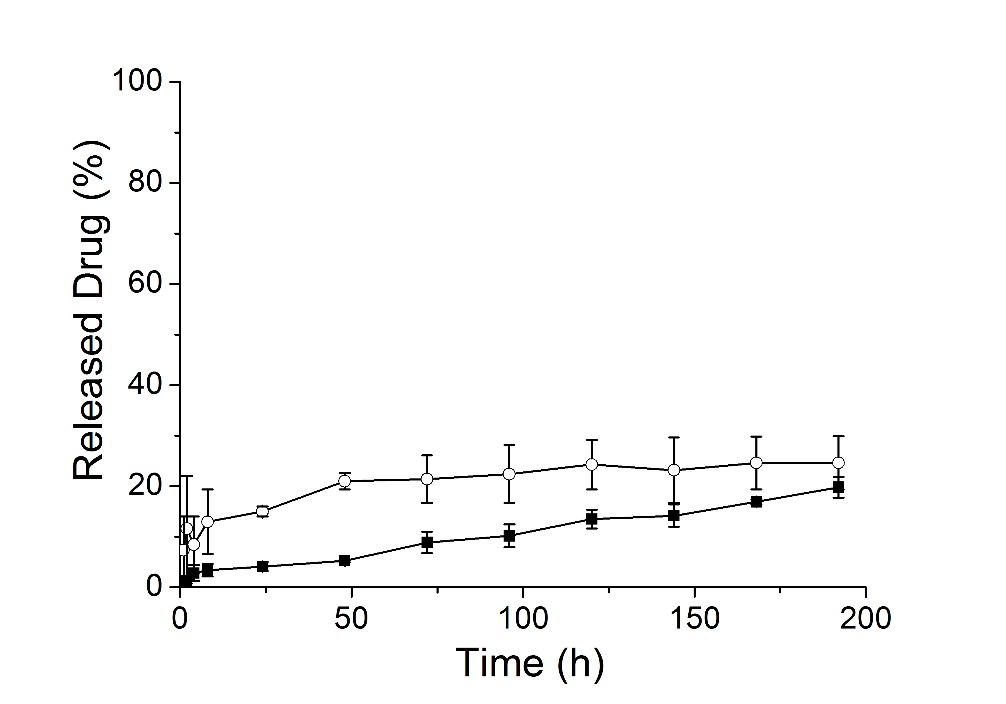
**

**Supplementary Figure S1. Physicochemical release kinetics of CSLPHNPs in neutral media.** Temporal release of FTY720 (black squares) and docetaxel (white circles) at pH 7.4 was measured by liquid chromatography-tandem mass spectrometry (LC-MS/MS). Points, mean of three experiments performed in triplicate. Bars, SE.

**
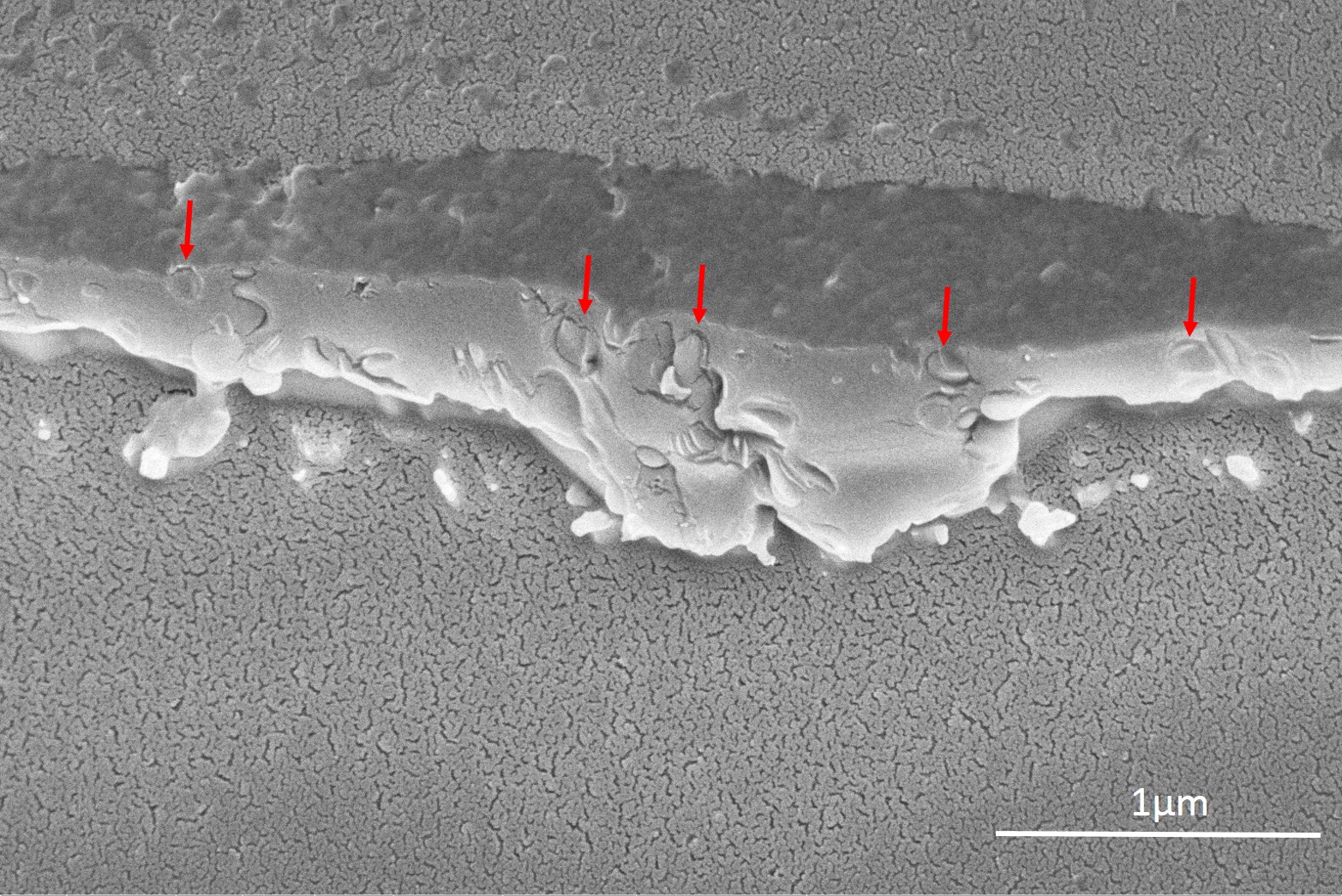

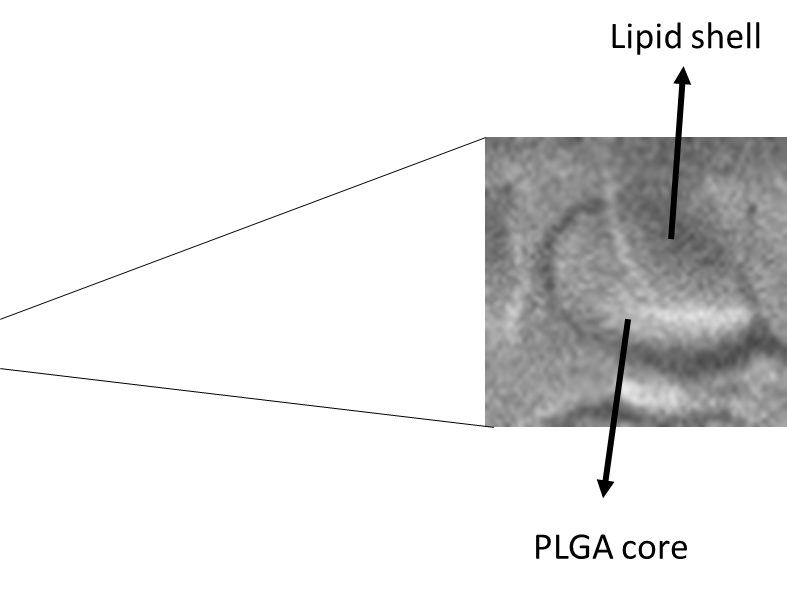
**

**Supplementary Figure S2. Nanoparticle physical structure determined by freeze-fracture scanning electron microscopy (FF-SEM).** Cryogenic sample was imaged at 3kV. Arrows indicate fractured CSLPHNPs, which demonstrate a core shell structure. Magnification: 100,000x. Scale bar indicates 1µm.

**
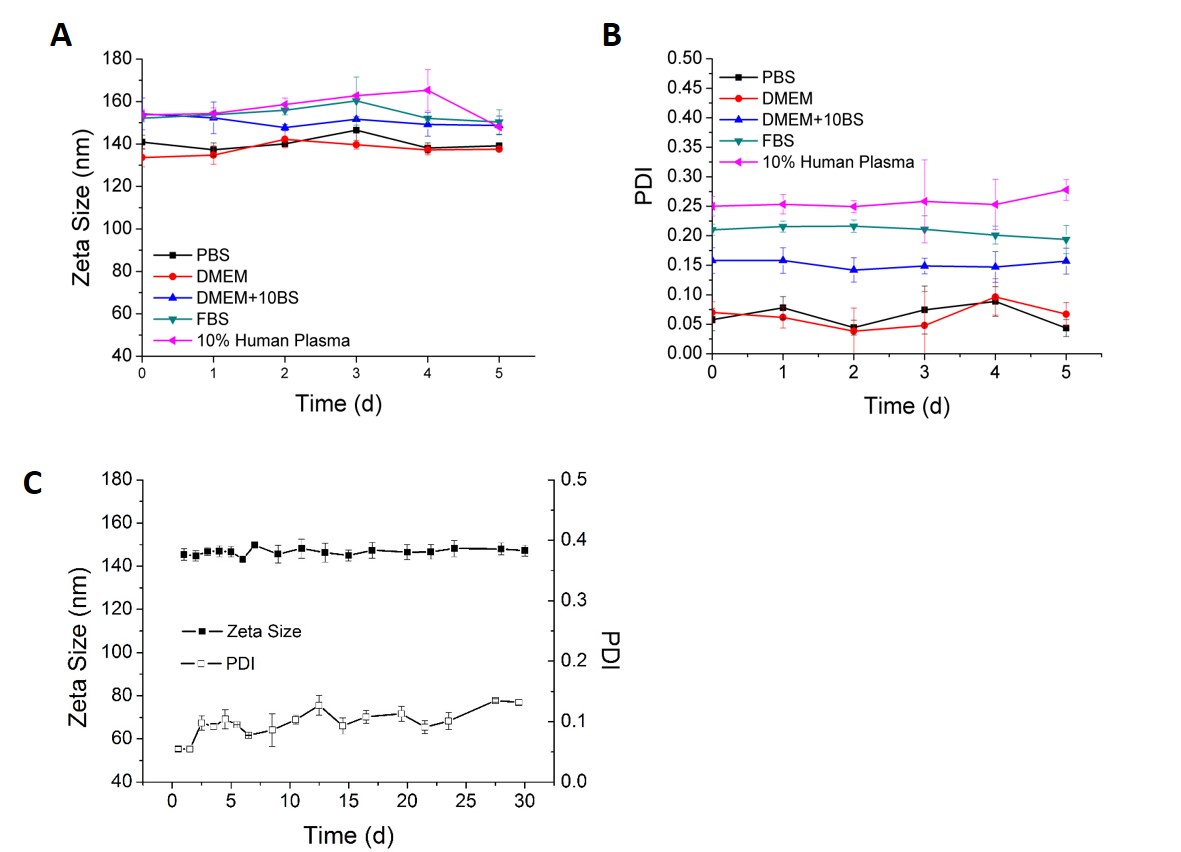
 Supplementary Figure S3. Long-term stability of the CSLPHNPs. A)** Size and **B)** polydispersity index (PDI) of CSLPHNPs in PBS, DMEM, DMEM+FBS, FBS, and human plasma, which were monitored for a period of 5 days using dynamic light scattering (DLS). **C)** Size and PDI measurements of CSLPHNPs suspended in PBS and stored at 4°C for up to 30 days. Data is expressed as Mean of three experiments ± SE.

**Supplementary Figure S4. Table of particle size, polydispersity index and zeta potential of CSLPHNPs.**

**
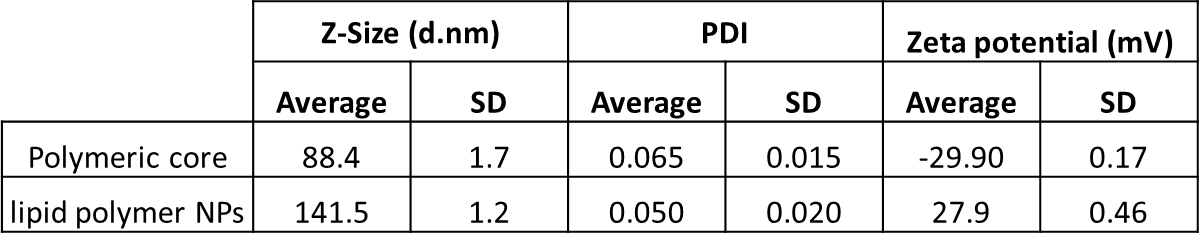
**

**
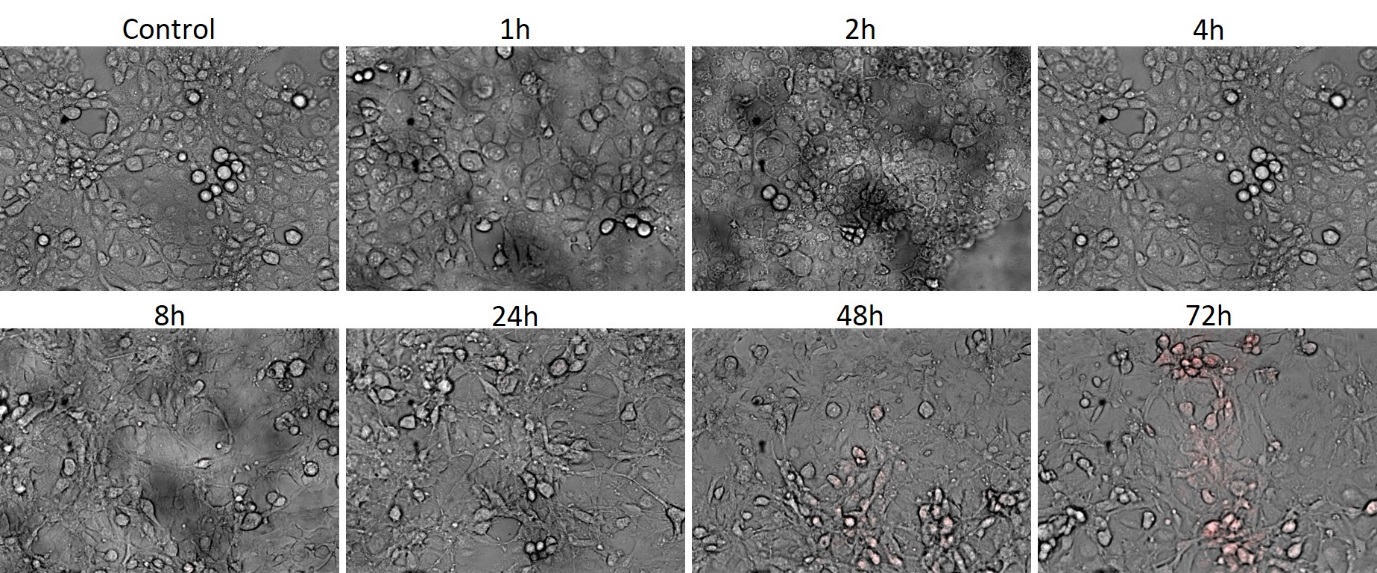
**

**Supplementary Figure S5. CSLPHNPs uptake in DU145 cell line.** Fluorescent/phase contrast images of hormone-refractory metastatic prostate cancer DU145 cells that were treated CSLPHNPs containing Rhodamine B for 72 hours.

**
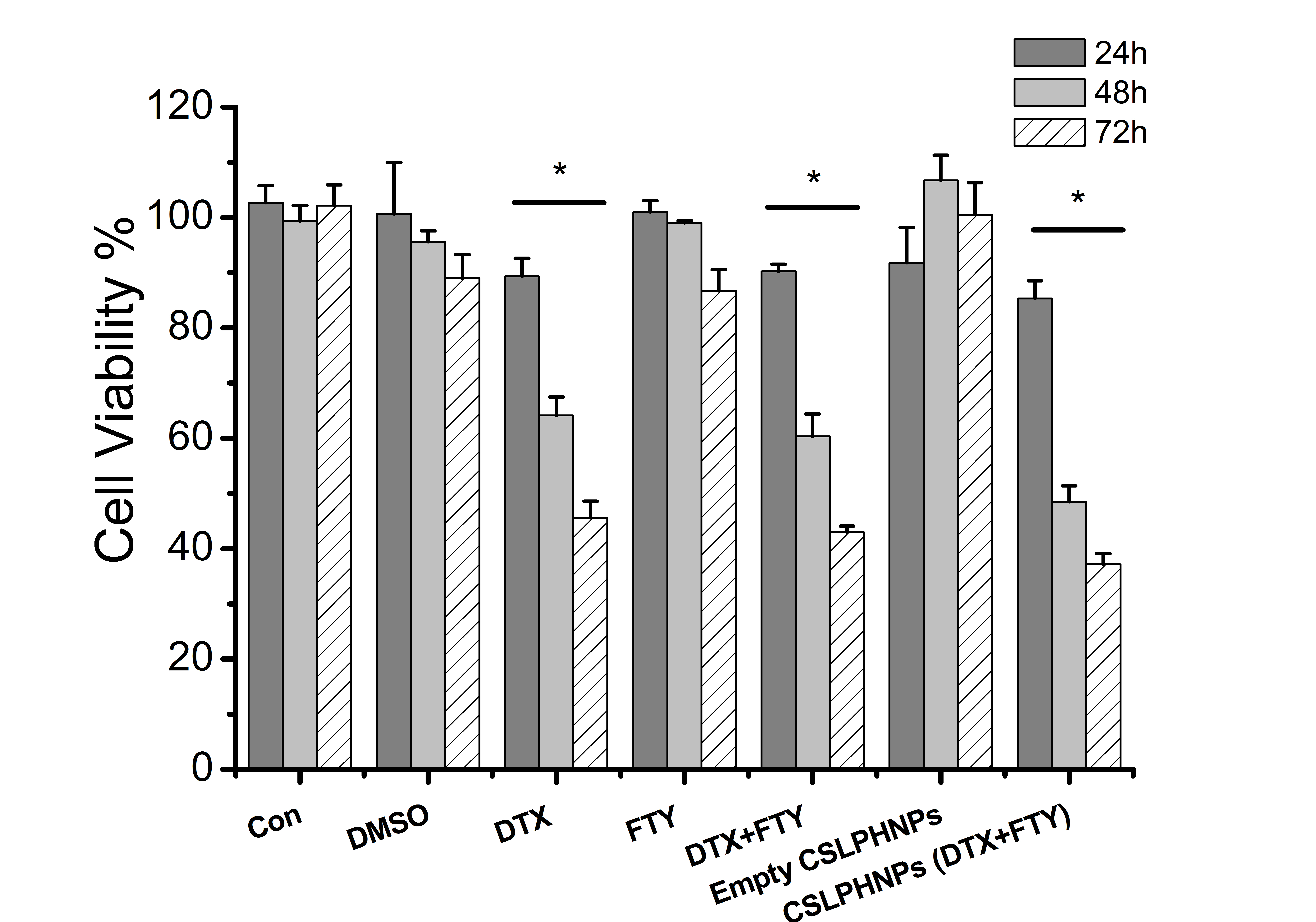
**

**Supplementary Figure S6. Cytotoxicity of CSLPHNPs on DU145 cell line.** Hormone-refractory metastatic prostate cancer DU145 cells were treated with free drugs or CSLPHNPs for 72 hours. Cytotoxicity of treatments was assessed using the MTT assay. Data is expressed as Mean ± SE, (n=4). *, *p*<0.05 vs. control.

**
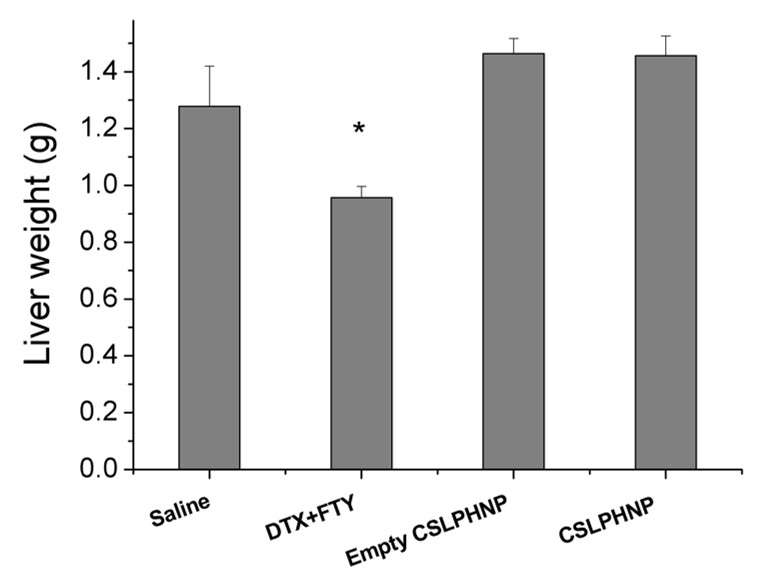
**

**Supplementary Figure S7. Evaluation of potential side effects of various drug formulations.** NSG immune deficient mice were injected with 1 × 10^6^ PC-3 cells and tumors were grown for 3 weeks. Mice were randomized in groups (n=7) according to treatment as shown in figure, and treated for 2 weeks every second day. At Day 30 mice were sacrificed and liver weight measurements were performed. Liver weight was normalized to control group (healthy group no treatment). Data is expressed as Mean (n=7) ± SE. *, *p* < 0.05 vs. control group.
